# Ziemssen’s Cyclopedia, vol. xvii

**Published:** 1879-01

**Authors:** 


*Cyclopædia of the Practice of Medicine.* Edited by Dr. H. VON ZIEMSSEN, Vol. XVII. General Anomalies of Nutrition and Poisons. ALBERT H. BUCK, M. D., Editor of American Edition, New York: Wm. Wood & Co.

Professor H. Immermann, of Basel, treats in this volume of Hemophilia Scurvy and Purpura Hemorrhagica. These subjects are presented with great care and completeness. Professor R. Boehm, of Dorpat, treats of poisoning by metalloids, acids, alkalies and their salts, also by anæsthetics, carbon compounds, and by tainted articles of diet. His sections upon chloroform and ether merit careful consideration. He records the facts as obtained in reference to the use of these agents, with scientific exactness, and those who make use of anæsthetic agents cannot be too familiar with these facts. The foot notes added by the American translator are well appended, and form with the text a true and safe practical guide to the practitioner. Professor Naunyn, of Kõnigsburg, treats of poisoning by the heavy metals and these salts. Professor H. Von Boeck, of Munich, treats of vegetable poisons.

The sections upon Arsenic, Atropine, Digitalis, Morphine and Ergot, are quite interesting to the reader, but we confess to a feeling that there is room for more thorough investigation of these subjects on which it is more desirable than ever, that precise knowledge should be obtained. It is quite desirable for instance, that the good and bad effects of digitalis and ergot should be well understood, and the signs by which these effects upon general nutrition and special facts may be early and speedily recognized. It is a good foundation laid for further precise investigation.

